# Palmitoylation-mediated synaptic regulation of AMPA receptor trafficking and function

**DOI:** 10.1007/s12272-019-01134-z

**Published:** 2019-03-05

**Authors:** Heesung Sohn, Mikyoung Park

**Affiliations:** 1 Center for Functional Connectomics, Brain Science Institute, Korea Institute of Science and Technology, Seoul, 02792 South Korea; 2 Department of Life Sciences, School of Natural Science, Hanyang University, Seoul, 04763 South Korea; 3 Department of Neuroscience, Korea University of Science and Technology, Daejeon, 34113 South Korea

**Keywords:** AMPAR trafficking, Palmitoylation, Synapse, Synaptic plasticity, Synaptic proteins, Neurodegenerative disease

## Abstract

The α-amino-3-hydroxy-5-methyl-4-isoxazolepropionic acid receptor (AMPAR) is a major glutamate-gated ion channel in the brain and is important for synaptic transmission, synaptic plasticity, and learning. Palmitoylation, a post-translational modification, is a critical process regulating AMPAR trafficking, synaptic function and plasticity, and learning and memory in health and diseases. In this review, we discuss current knowledge on the palmitoylation-dependent regulation of AMPAR trafficking and functions. We focus on the palmitoylation of AMPARs and other synaptic proteins that directly or indirectly interact with AMPARs, including postsynaptic density 95, glutamate receptor-interacting protein/AMPAR-binding protein, A-kinase anchoring protein 79/150, and protein interacting with C kinase 1. Finally, we discuss what future studies should address in the field of palmitoylation-dependent AMPAR trafficking and function with regard to physiology and neurodegenerative diseases.

## Introduction

Synapses are the fundamental bases of brain structure and function and consist of a presynaptic terminal, synaptic cleft, and postsynaptic site. Neurons communicate with each other by releasing neurotransmitters from presynaptic terminals to synaptic clefts, detecting neurotransmitters with postsynaptic neurotransmitter receptors, and translating the detection into electrical and chemical signals in the postsynaptic cells. In addition to synaptic transmission, synapses also possess a remarkable ability to modulate their outputs in response to various synaptic inputs, which is called synaptic plasticity. The most well characterized examples of synaptic plasticity in the brain are long-term potentiation (LTP) and long-term depression (LTD).

The α-amino-3-hydroxy-5-methyl-4-isoxazolepropionic acid receptor (AMPAR), a major glutamate-gated ion channel in the mammalian central nervous system, play important roles in synaptic transmission, synaptic plasticity such as LTP and LTD, and ultimately, learning and memory cognitive function in the brain. The synaptic localization and abundance of AMPARs are critical for synaptic function and are regulated by exocytosis, endocytosis, recycling, and lateral mobility on the surface membranes of AMPARs (Lu et al. 2001; Borgdorff and Choquet 2002; Tardin et al. 2003; Park et al. 2004; Heine et al. 2008; Patterson et al. 2010; Wu et al. 2017; Park 2018). Post-translational modifications (PTMs), including phosphorylation (Banke et al. 2000; Lee et al. 2000, 2003, 2010; Lu and Roche 2012; Lussier et al. 2015), glycosylation (Hollmann et al. 1994; Standley and Baudry 2000; Traynelis et al. 2010), ubiquitination (Schwarz et al. 2010; Lussier et al. 2011, 2012; Widagdo et al. 2015; Wei et al. 2016), sumoylation (Craig and Henley 2012; Jaafari et al. 2013; Lee et al. 2013), and palmitoylation (Hayashi et al. 2005; Lin et al. 2009; Thomas et al. 2012, 2013; Diering and Huganir 2018), are also critical regulatory factors for AMPAR function, synaptic plasticity, and learning and memory in health and diseases. Among these PTMs, palmitoylation, which was the most frequently observed in neuronal cells than other lipid modifications and was demonstrated to play crucial roles in synaptic function and neurodegenerative diseases (Fukata and Fukata 2010; Cho and Park 2016), will be discussed in this review.

Palmitoylation is a lipid modification that occurs by a covalent attachment of the 16 carbon-containing saturated fatty acid palmitate to specific cysteine (Cys) residues of target proteins via a thioester bond. This process is catalyzed by conserved Aspartate-Histidine-Histidine-Cys (DHHC) motif-containing palmitoyl acyltransferases (PATs), also known as DHHC enzymes because of their catalytic DHHC motif, and reversed by depalmitoylating enzymes such as acyl protein thioesterases and palmitoyl protein thioesterases. The reversibility of palmitoylation enables precise and dynamic regulation of protein functions in neurons, including synaptic targeting, glutamate receptor trafficking, synaptic transmission, and synaptic plasticity (Kang et al. 2004, 2008; Hayashi et al. 2009; Lin et al. 2009; Noritake et al. 2009; Keith et al. 2012; Thomas et al. 2012). Further, the activities of a variety of PATs and depalmitoylating enzymes have been linked to many neurodegenerative diseases (Han et al. 2015; Cho and Park 2016).

Given the importance of AMPARs and palmitoylation in the physiology of synaptic function, synaptic plasticity and learning, we present the current knowledge on palmitoylation-mediated synaptic regulation of AMPARs and their interacting proteins and discuss possible future studies on palmitoylation-dependent AMPAR trafficking in neurodegenerative diseases in this review. The interacting proteins discussed include postsynaptic density 95 (PSD-95), glutamate receptor-interacting protein (GRIP1)/AMPAR-binding protein (ABP), A-kinase anchoring protein 79/150 (AKAP79/150), and protein interacting with C kinase 1 (PICK1).

## AMPAR palmitoylation

**Fig. 1:**
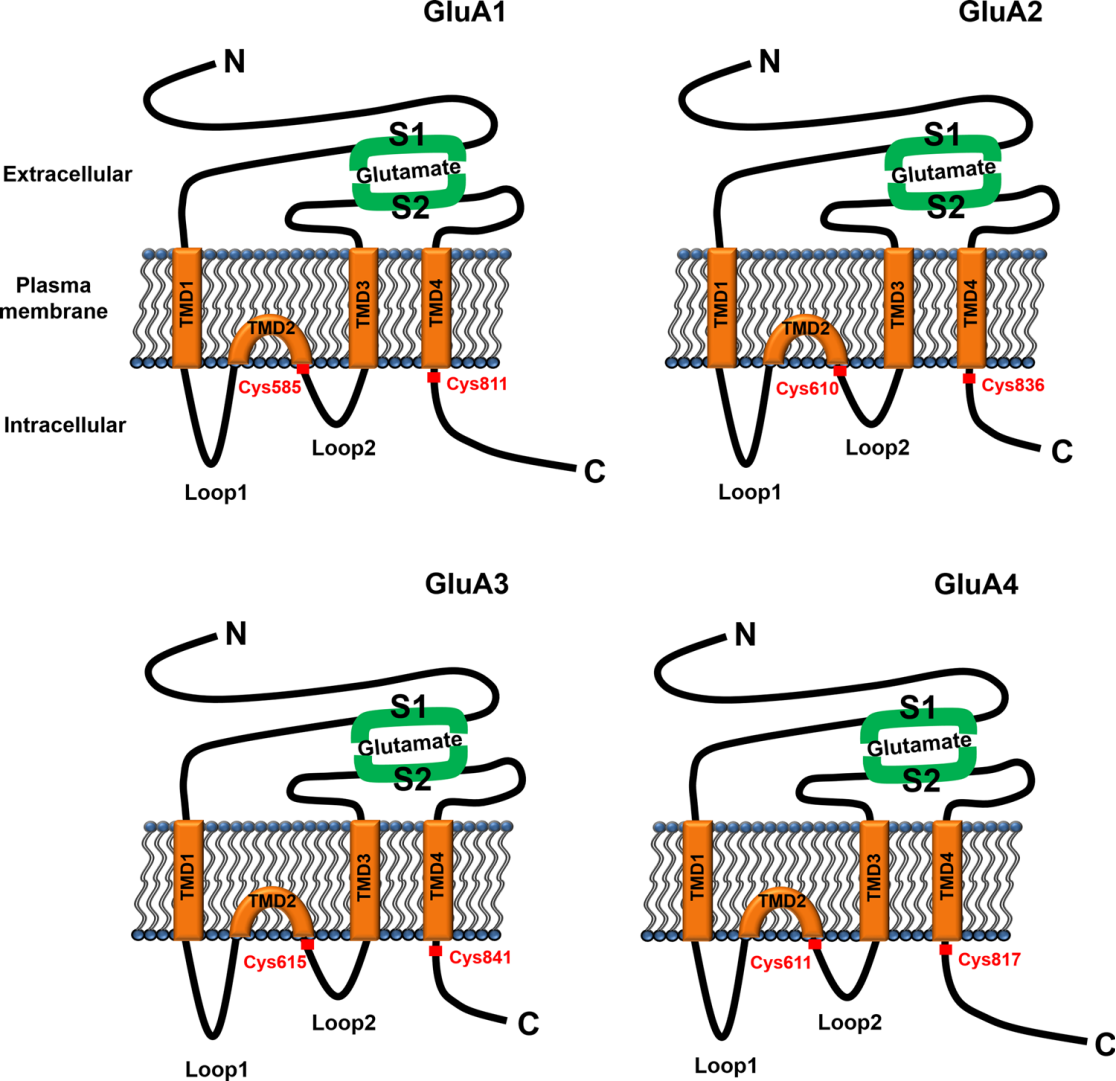
α-Amino-3-hydroxy-5-methyl-4-isoxazolepropionic acid receptor (AMPAR) structure and palmitoylation. Schematic diagrams show each of the four AMPAR subunits. In each diagram, the large extracellular N-terminal domain includes S1, which forms the glutamate binding site together with S2 that is located on the extracellular loop linking transmembrane domain 3 (TMD3) and TMD4. Four hydrophobic TMDs including three membrane-spanning TMDs (TMD1, TMD3, and TMD4) and one membrane-embedded TMD (TMD2) and three intracellular domains (intracellular loop1, loop2, and the cytoplasmic tail) are shown. Palmitoylation sites for each subunit are marked in red

AMPARs are heterotetrameric proteins composed of the subunits GluA1-GluA4 (also called GluR1-GluR4 or GluRA-GluRD) (Wisden and Seeburg 1993; Hollmann and Heinemann 1994; Dingledine et al. 1999; Collingridge et al. 2009; Traynelis et al. 2010). In the hippocampus, the composition of AMPARs is developmentally distinct (Wenthold et al. 1996; Zhu et al. 2000), with GluA1/GluA2 heteromeric AMPARs more predominant than GluA2/GluA3 heteromers in mature hippocampal neurons (Wenthold et al. 1996; Lu et al. 2009). Nevertheless, all four AMPAR subunits have similar structures and topologies (Malinow and Malenka 2002; Lu and Roche 2012). Each subunit has a large extracellular N-terminal domain, four hydrophobic transmembrane domains (TMDs), including three that are membrane spanning (TMD1, TMD3, and TMD4) and the membrane-embedded TMD2 that contributes to channel pore formation, and three intracellular domains (loop1, loop2, and a C-terminal tail) (Fig. 1). Although the N-terminal domain and four hydrophobic TMDs are highly homologous among the subunits, their C-terminal intracellular tails are distinct, which confer distinct regulation to the AMPAR subunits through specific interactions with cytoplasmic proteins (Malinow and Malenka 2002; Shepherd and Huganir 2007).

**Fig. 2:**
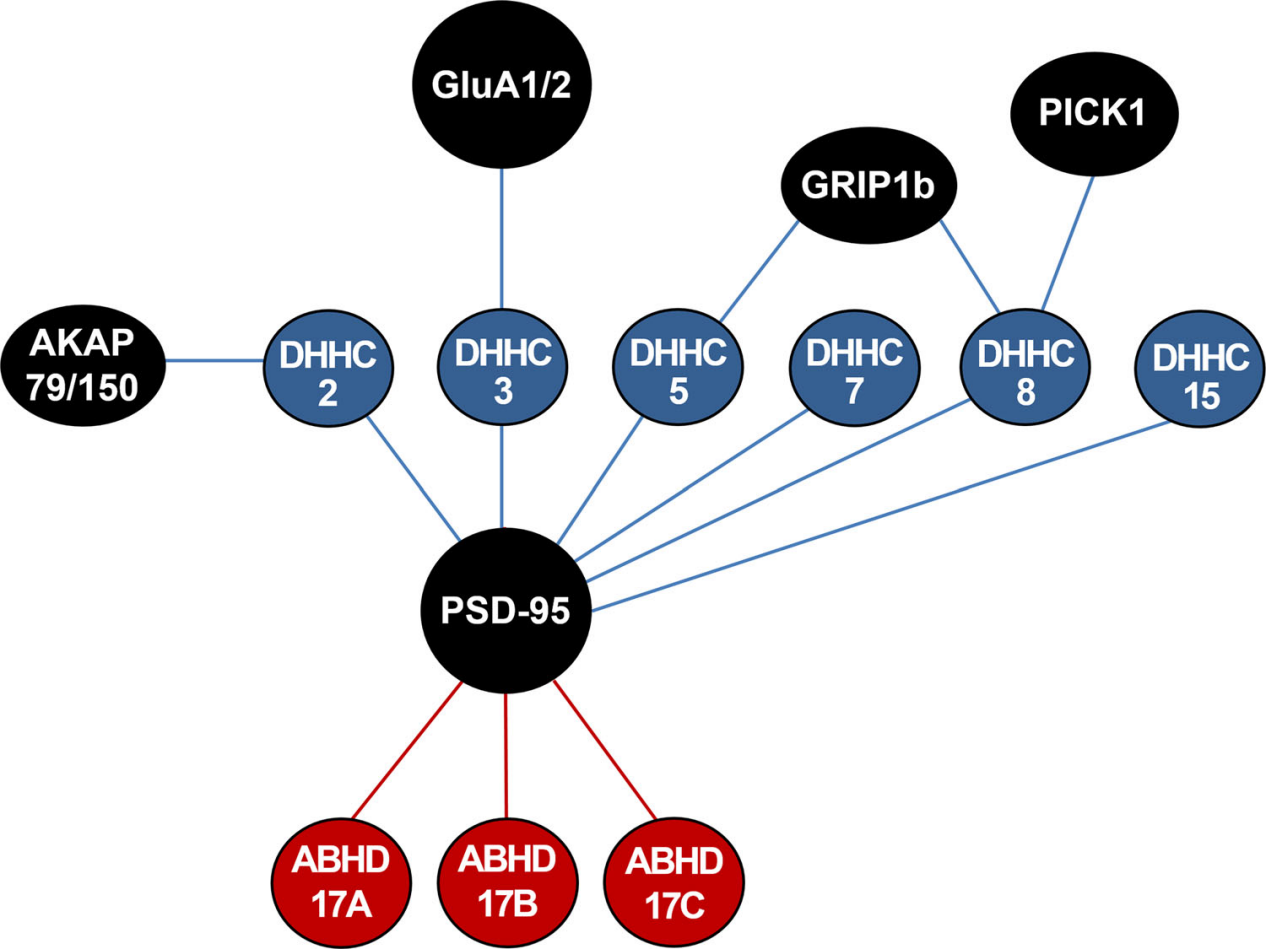
Interaction network between palmitoyl acyltransferases (PATs)/depalmitoylating enzymes and their synaptic substrates. Only the PAT/depalmitoylating enzyme–substrate pairs described in this review are shown. Blue circles, PAT enzymes; red circles, depalmitoylating enzymes; black circles, synaptic substrates. PSD-95, postsynaptic density 95; GRIP1, glutamate receptor-interacting protein; AKAP79/150, A-kinase anchoring protein 79/150; PICK1, protein interacting with C kinase 1; ABHD17, α/β-hydrolase domain-containing protein 17

All four AMPAR subunits are palmitoylated at two conserved Cys residues: one immediately next to TMD2, and the other next to TMD4 in the C-tail juxtamembrane region (labeled red in Fig. 1) (Hayashi et al. 2005; Diering and Huganir 2018). Palmitoylation at Cys585 in GluA1 and at Cys610 in GluA2 immediately after TMD2, which is mediated by a PAT, DHHC3 (also known as Golgi-specific DHHC zinc finger protein [GODZ]; (Uemura et al. 2002) (Fig. 2), accumulated AMPARs in the Golgi apparatus and reduced AMPAR surface expression (Hayashi et al. 2005). The finding of accumulation of palmitoylated AMPARs at the Golgi apparatus implies that depalmitoylation of AMPARs at the Golgi apparatus would be a releasing signal for AMPARs to traffic forward to the plasma membrane. Palmitoylation at Cys811 in GluA1 and at Cys836 in GluA2 in the C-tail juxtamembrane region did not affect the AMPAR surface expression level at steady state. Rather, it inhibited the interaction of GluA1 with the 4.1N protein and regulated activity-dependent endocytosis of GluA1 (Hayashi et al. 2005), the insertion of GluA1 into the plasma membrane, and LTP (Lin et al. 2009). Interestingly, GluA1 palmitoylation requires anterograde trafficking from the endoplasmic reticulum (ER) to the Golgi apparatus, where DHHC3/GODZ is located (Uemura et al. 2002), whereas GluA2 palmitoylation primarily occurs within the ER (Yang et al. 2009). Because DHHC3/GODZ was reported to be exclusively located on the Golgi apparatus (Uemura et al. 2002), the PAT in the ER that palmitoylates GluA2 remains to be revealed. In addition, because palmitoylation next to TMD2 at Cys585 in GluA1 and at Cys610 in GluA2 occurred only when co-expressed with DHHC3/GODZ (Hayashi et al. 2005), it is very plausible that other PATs palmitoylate AMPAR residues immediately after TMD2 endogenously or that DHHC3/GODZ or other PATs palmitoylate AMPAR residues when some stimuli are applied.

Neuronal activity also regulates AMPAR palmitoylation dynamically (Hayashi et al. 2005; Yang et al. 2009; Spinelli et al. 2017). Glutamate treatment, which stimulates neurons, induced depalmitoylation of AMPARs (Hayashi et al. 2005; Yang et al. 2009) whereas tetrodotoxin (TTX) treatment, which blocks neuronal activity, enhanced palmitoylation of AMPARs (Yang et al. 2009). Interestingly, a high-fat diet elevated levels of palmitic acid and insulin resistance, which led to increased expression of DHHC3/GODZ in the hippocampus (Spinelli et al. 2017). Elevated levels of palmitic acid and DHHC3/GODZ caused GluA1 palmitoylation, which suppressed its activity-dependent delivery to the plasma membrane and impaired LTP and memory in mice fed a high-fat diet (Spinelli et al. 2017). In addition to the studies on AMPAR palmitoylation in hippocampal and cortical neurons (Hayashi et al. 2005; Lin et al. 2009; Yang et al. 2009; Spinelli et al. 2017), a study performed in the nucleus accumbens demonstrated that intraperitoneal administration of cocaine enhanced palmitoylation of GluA1 and GluA3 and redistributed intracellular GluA1 and GluA3 (Van Dolah et al. 2011). Cocaine-induced palmitoylation and subcellular redistribution of GluA1 and GluA3 were blocked by the application of the palmitoylation inhibitor 2-bromopalmitate (Van Dolah et al. 2011). These findings suggest that the differential roles of AMPAR palmitoylation in various forms of synaptic plasticity should be investigated further in multiple brain regions.

## Regulation of AMPARs by palmitoylation of synaptic proteins

In addition to the direct palmitoylation of AMPARs themselves, palmitoylaton of synaptic proteins directly or indirectly interacting with AMPARs (Kim and Sheng 2004) such as PSD-95, GRIP1, GRIP2, PICK1, and AKAP79/150, can also be an important regulatory factor for AMPAR trafficking and function (Fukata and Fukata 2010; Thomas and Huganir 2013; Han et al. 2015).

**Fig. 3:**
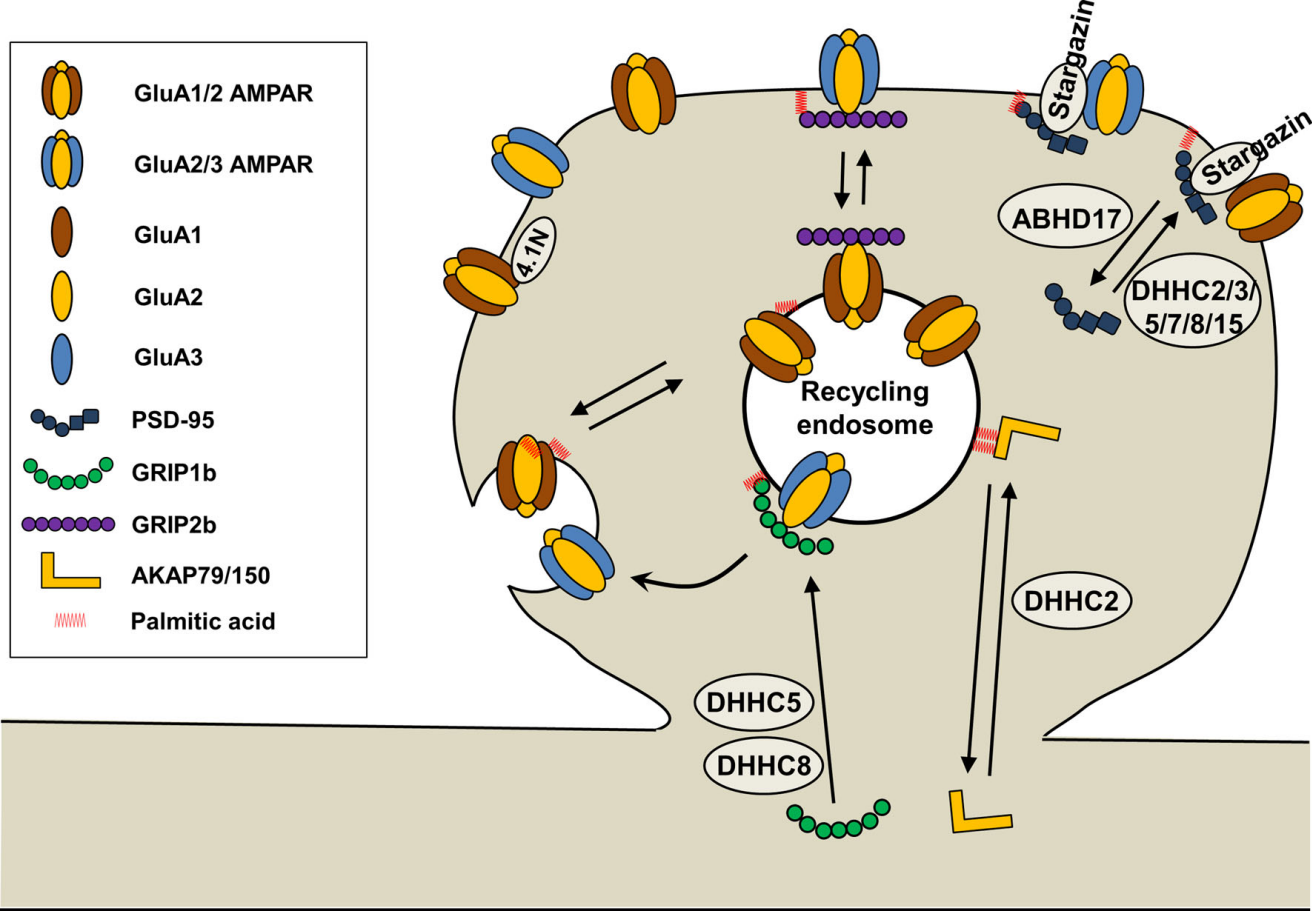
AMPAR palmitoylation and regulation of AMPARs by palmitoylation of synaptic proteins. Palmitoylation at Cys811 in the GluA1 C-terminus inhibits the interaction of GluA1 with 4.1N and triggers activity-dependent endocytosis of AMPARs. PSD-95 palmitoylations at Cys3 and Cys5 mediated by DHHC2, DHHC3, DHHC5, DHHC7, DHHC8, and DHHC15, stabilize PSD-95-AMPAR interaction via Stargazin. GRIP1b palmitoylation targets GRIP1b to recycling endosomes and enhances activity-dependent recycling of GluA2-containing AMPARs to the plasma membrane. AKAP79 palmitoylations at Cys36 and Cys129 mediated by DHHC2 are necessary for AKAP79 targeting to recycling endosomes and dendritic spines. PSD-95, postsynaptic density 95; GRIP1, glutamate receptor-interacting protein; AKAP79/150, A-kinase anchoring protein 79/150

PSD-95, the major PSD scaffolding protein, regulates synaptic trafficking of AMPARs (El-Husseini et al. 2000; Elias and Nicoll 2007). PSD-95 is palmitoylated at Cys3 and Cys5 by DHHC2, DHHC3/GODZ, DHHC5, DHHC7, DHHC8, and DHHC15 (Fukata et al. 2004) (Figs. 2, 3), which is required for its synaptic targeting (Topinka and Bredt 1998; Craven et al. 1999; El-Husseini et al. 2000; Noritake et al. 2009; Sturgill et al. 2009) and for the synaptic trafficking of AMPARs (El-Husseini et al. 2000; El-Husseini Ael et al. 2002; Schnell et al. 2002; Noritake et al. 2009). Overexpression of PSD-95 increased AMPAR-mediated synaptic transmission (El-Husseini et al. 2000; Schnell et al. 2002) whereas the non-palmitoylatable form of PSD-95 disrupted GluA1 clustering and AMPAR-mediated synaptic transmission (El-Husseini et al. 2000; El-Husseini Ael et al. 2002; Schnell et al. 2002). Glutamate treatment (10 µM) caused PSD-95 depalmitoylation, PSD-95 synaptic cluster dispersal, and AMPAR internalization (El-Husseini Ael et al. 2002). Ca^2+^/calmodulin (CaM) binding to the N-terminus of PSD-95 was shown to block PSD-95 palmitoylation at Cys3 and Cys5, which triggered PSD-95 dispersal and dissociation from the postsynaptic membrane (Zhang et al. 2014). Conversely, treatment with neuronal activity blockers such as kynurenic acid (an ionotropic glutamate receptor blocker), APV (an N-methyl-d-aspartate receptor [NMDAR] blocker), CNQX (an AMPAR blocker), and TTX (a sodium channel blocker) increased the palmitoylation and synaptic clustering of PSD-95 (Noritake et al. 2009) as TTX increased AMPAR palmitoylation (Yang et al. 2009). This activity-dependent palmitoylation of PSD-95 is mediated by DHHC2, which is translocated from the dendritic shaft to the PSD upon activity blockade (Noritake et al. 2009). The palmitoylation of PSD-95 altered its conformation from a compact to an extended form, which was oriented perpendicular to the PSD membrane (Jeyifous et al. 2016) and associated indirectly with AMPARs via Stargazin, an AMPAR auxiliary protein (Tomita et al. 2005) (Fig. 3), or NMDARs via the GluN2B subunit. Increased PSD-95 palmitoylation also increased the surface AMPAR level with no change in the NMDAR level, indicating the differential regulation of AMPAR and NMDAR trafficking by PSD-95 palmitoylation (Jeyifous et al. 2016).

In addition to controlling the PSD-95 palmitoylation process, direct regulation of the PSD-95 depalmitoylation process is definitely another key regulatory step for synaptic function. Recently, long-awaited PSD-95 depalmitoylating enzymes were identified as α/β-hydrolase domain-containing protein 17 members (ABHD17A, ABHD17B, and ABHD17C) (Yokoi et al. 2016) (Figs. 2, 3). Overexpression of ABHD17B selectively depalmitoylated PSD-95 and decreased the synaptic clustering of PSD-95 and GluA1 (Yokoi et al. 2016). Further investigations on PSD-95 deplamitoylation and palmitoylation mechanisms will help clarify the regulatory mechanisms underlying synaptic function and plasticity.

GRIP1 and GRIP2 (also known as ABP), which have multi-PSD-95/discs large/zona occludens (PDZ) domains, directly interact with the C-termini of the AMPAR GluA2 and GluA3 subunits through its PDZ and stabilize AMPARs (Dong et al. 1997; Srivastava et al. 1998) (Fig. 3). The genes encoding GRIP1 and GRIP2 have multiple splice isoforms (Dong et al. 1997; Wyszynski et al. 1999), and GRIP1b and GRIP2b (also known as pABP-long [pABP-L]) have additional N-terminal sequences, which contain a unique Cys residue that is palmitoylated (Yamazaki et al. 2001; DeSouza et al. 2002; Thomas et al. 2012). Palmitoylatable pABP-L targets the plasma membrane of dendritic spines where it associates with surface GluA2, whereas the non-palmitoylatable ABP-L form (a variant of ABP with seven PDZ domains) localizes with intracellular AMPARs (DeSouza et al. 2002; Misra et al. 2010) (Fig. 3). In addition, palmitoylated pABP-L increased the amplitude and frequency of AMPAR-mediated excitatory postsynaptic currents (Misra et al. 2010). Palmitoylation-deficient pABP-L that is mutated at the 11th amino acid Cys (to alanine) changed its localization to intracellular clusters from the spinal plasma membrane, indicating the requirement of pABP-L palmitoylation in its synaptic localization (DeSouza et al. 2002). Whereas the palmitoylation of GRIP2/ABP induced plasma membrane targeting, the palmitoylation of GRIP1b, which is mediated by DHHC5 and DHHC8 (Thomas et al. 2012) (Figs. 2, 3), targeted intracellular endosomes and enhanced NMDA-induced AMPAR endocytic recycling trafficking. This finding indicates the involvement of GRIP1 palmitoylation in AMPAR endocytic recycling during NMDA-induced LTD (Hanley and Henley 2010; Thomas et al. 2012) (Fig. 3). Although the PATs for GRIP1b, DHHC5 and DHHC8 were identified (Thomas et al. 2012), the PATs for GRIP2b remain to be discovered.

AKAP79/150 (AKAP79 in humans and AKAP150 in rodents), a scaffold protein that is not a direct binding protein with AMPARs, is palmitoylated at Cys36 and Cys129 in AKAP79 by DHHC2 (Keith et al. 2012; Delint-Ramirez et al. 2015; Woolfrey et al. 2015) (Figs. 2, 3). Palmitoylation of AKAP79/150 is required for its targeting to recycling endosomes (Keith et al. 2012; Purkey et al. 2018) and dendritic spines upon LTP-inducing stimulation (Keith et al. 2012; Woolfrey et al. 2015). Conversely, LTD-inducing stimulation, which is accompanied by spine shrinkage, required depalmitoylation and synaptic removal of AKAP79/150 (Keith et al. 2012; Woolfrey et al. 2018). Super-resolution (approximately 40–60 nm) stimulated emission depletion microscopy revealed that palmitoylation-deficient AKAP150 localization to the PSD was significantly reduced in AKAP150 palmitoylation-deficient knockin mice (Purkey et al. 2018). In addition, AKAP150 palmitoylation restricted synaptic localization of Ca^2+^-permeable AMPARs containing GluA1 but lacking GluA2 in the basal state and was required for Ca^2+^-permeable AMPAR-dependent LTP (Purkey et al. 2018).

In addition to PSD-95, GRIP/ABP, and AKA79/150, many other synaptic proteins have also been reported to be palmitoylated (Fukata and Fukata 2010; Cho and Park 2016). PICK1 is a PDZ domain-containing protein that directly interacts with GluA2 and GluA3 (Dev et al. 1999; Xia et al. 1999). PICK1 plays critical roles in cerebellar LTD (Steinberg et al. 2006; Anggono et al. 2013; Thomas et al. 2013), hippocampal synaptic plasticity (Terashima et al. 2004, 2008; Volk et al. 2010; Anggono et al. 2011) and cortical synaptic plasticity (Clem et al. 2010) by regulating synaptic abundance, trafficking, and functions of AMPARs. Palmitoylation of PICK1 by DHHC8 was reported to be critical for cerebellar LTD (Thomas et al. 2013). Even though important work has reported the roles of PICK1 in AMPAR trafficking, synaptic plasticity, and learning and memory, very little is known about how PICK1 is palmitoylated and how this contributes to AMPAR trafficking and synaptic function.

## Perspectives

Our understanding of the roles of palmitoylation in synaptic targeting, glutamate receptor trafficking, synaptic transmission, synaptic plasticity, and learning and memory has increased with the aid of identifications of pamitoylating enzymes, namely, the DHHC family PATs (Fukata et al. 2004; Fukata and Fukata 2010) and depalmitoylating ABHD enzymes (Yokoi et al. 2016) and development of the non-radioactive palmitoylation assay (Wan et al. 2007) and omics technologies (Kang et al. 2008). Many neuronal substrates for PATs have been discovered with rather broad specificities of the PATs as well as the substrates (Fukata and Fukata 2010; Cho and Park 2016). Future investigations will be required to clarify specific substrate and PAT pairs by dissecting the mechanisms underlying these specificities. In addition, the detailed mechanisms underlying how PATs regulate synaptic function in pathophysiology remain to be revealed. Furthermore, depalmitoylation-mediated regulation of synaptic function remains largely unexplored.

Palmitoylation of AMPARs is affected by another PTM, namely phosphorylation (Lin et al. 2009). Depalmitoylation at Cys811 in the C-tail of GluA1 positively regulates nearby protein kinase C phosphorylation at Ser816 and Ser818, which enhances the interaction of 4.1N with GluA1, and facilitates GluA1 insertion and LTP (Lin et al. 2009). Given that ubiquitination has also been suggested to modulate signaling for fine regulation of AMPAR trafficking via communication with palmitoylation (Yang et al. 2009) or phosphorylation of GluA1 (Kessels et al. 2009), and that synaptic targeting of PSD-95 is also reciprocally controlled by nitrosylation and palmitoylation (Ho et al. 2011), it will be important to further dissect how AMPAR trafficking and synaptic function are regulated through crosstalk among PTMs.

Protein palmitoylation is impaired in various neurodegenerative diseases including Alzheimer’s disease (Mizumaru et al. 2009; Bhattacharyya et al. 2013), Huntington’s disease (Singaraja et al. 2002; Huang et al. 2004; Yanai et al. 2006; Singaraja et al. 2011; Milnerwood et al. 2013), schizophrenia (Liu et al. 2002; Mukai et al. 2004, 2008; Mukai et al. 2015), intellectual disability (Mansouri et al. 2005; Tarpey et al. 2009; Masurel-Paulet et al. 2014; Mitchell et al. 2014), and neuronal ceroid lipofuscinosis (Vesa et al. 1995; Henderson et al. 2016). Altered AMPAR trafficking has also been described in neurological disorders (Ikonomovic et al. 1995, 1997; Muddashetty et al. 2007; Suvrathan et al. 2010; Reinders et al. 2016; Jurado 2017). To our knowledge, however, studies showing direct evidence of palmitoylation-mediated regulation of AMPAR trafficking in neurological diseases are absent. Future studies detailing how the palmitoylation and depalmitoylation of AMPARs and other synaptic proteins affect the pathogeneses of neurodegenerative diseases are needed.

AMPAR trafficking, especially the surface delivery of AMPARs, is crucial for the physiological functioning of synapses and pathogeneses of neurodegenerative diseases. As described in this review, AMPARs themselves and other AMPAR-interacting neuronal proteins are palmitoylated, and palmitoylation plays important roles in the surface delivery of AMPARs, which is necessary for synaptic function. Therefore, future studies in the field of neuronal palmitoylation should focus on the regulation of AMPAR trafficking by specific PATs and depalmitoylating enzymes in pathophysiological conditions. This will provide informative clues for the development of selective pharmacological therapeutics aimed at ameliorating neurodegenerative diseases derived from PATs or depalmitoylating enzymes associated with AMPAR trafficking.
